# The Stresses and Deformations in the Abfraction Lesions of the Lower Premolars Studied by the Finite Element Analyses: Case Report and Review of Literature

**DOI:** 10.3390/diagnostics14080788

**Published:** 2024-04-09

**Authors:** Bogdan Constantin Costăchel, Anamaria Bechir, Mihail Târcolea, Lelia Laurența Mihai, Alexandru Burcea, Edwin Sever Bechir

**Affiliations:** 1 Doctoral School in Dental Medicine, “Titu Maiorescu” University of Bucharest, 189 Calea Văcăreşti, 040056 Bucharest, Romania; costachel.bogdan@gmail.com; 2 Faculty of Dental Medicine, “Titu Maiorescu” University of Bucharest, 67A Gh. Petrascu Street, 031592 Bucharest, Romania; lelia_mihai2000@yahoo.com (L.L.M.); alexandru.burcea@helpdent.ro (A.B.); 3 Faculty of Materials Science and Engineering, University Politehnica of Bucharest, 313 Splaiul Independenţei, 060042 Bucharest, Romania; 4 Faculty of Dental Medicine, “George Emil Palade” University of Medicine, Pharmacy, Science, and Technology of Targu Mures, 38 Gh. Marinescu Street, 540142 Targu Mures, Romania; bechir.edwin@gmail.com

**Keywords:** mandibular premolars, abfraction lesions, FEA, experimental forces, evaluation

## Abstract

Background: The purpose of the study was to investigate the behavior of hard dental structures of the teeth with abfraction lesions when experimental occlusal loads were applied. Methods: A 65-year-old patient came to the dentist because she had painful sensitivity in the temporomandibular joints and the lower right premolars. The patient was examined, and cone-beam computed tomography (CBCT) of the orofacial area was indicated. The data provided from the CBCT were processed with Mimics Innovation Suite 17 software to create the desired anatomical area in 3D format. Then, the structural calculation module was used in order to perform a finite element analysis of the lower right premolar teeth. A focused review of articles published between 2014 and 2023 from specialty literature regarding the FEA of premolars with abfraction lesions was also conducted. Results: The parcel area and the cervical third of the analyzed premolars proved to be the most vulnerable areas under the inclined direction of occlusal loads. The inclined application of experimental loads induced 3–4 times higher maximum shears, stresses, and deformations than the axial application of the same forces. Conclusions: FEA can be used to identify structural deficiencies in teeth with abfractions, a fact that is particularly important during dental treatments to correct occlusal imbalances.

## 1. Introduction

The complex formed by periodontal tissues (cementum, ligaments, and bone) confers the characteristic flexibility of the tooth root in the alveolar socket, flexibility necessary for dispersing the occlusal forces [1,2,3]. Irreversible structural defects that are located in the cervical area at the level of the enamel–cement junction on the buccal/labial surface of the teeth are named non-carious cervical lesions (NCCLs) [4]. In the area of the enamel–cementum junction, the enamel prisms have a horizontal orientation [5]. The volume and composition of enamel and dentin contribute to the development of NCCLs [6]. 

The term “abfraction” nominates stress-induced NCCLs in the cervical region of the dental crowns that determine the progressive damage to mineralized dental tissues because of flexion [7,8]. Micro-fractures of hydroxyapatite (HA) crystals of enamel, cementum, and dentine thus appear in the zone of stress concentration. Abfraction lesions are located at the site of the least resistance in the cervical region, at the enamel–cementum junction [7,8,9]. 

The etiology of abfraction lesions remains disputable, and current evidence shows that the apparition of NCCLs is multifactorial, and that the degrees of tooth hard tissue vary and may differ from patient to patient [7,10,11,12,13]. Correct diagnosis and treatment decisions require in-depth knowledge regarding the multifactorial etiological conditions of abfraction lesions. The therapeutic possibilities for these lesions currently vary; therefore, clinicians need to be informed regarding the major etiological factors and the clinical particularities that differentiate them [7,14]. 

Computational modeling is a computer-based method that studies the behavior of complex systems through computer simulations. It can be used to resolve mathematical models that characterize physical phenomena but also to make predictions of the system’s behavior under different conditions [15,16]. Finite element analysis (FEA) is a numerical technique that slices the structure of the analyzed object into several elements; later, the elements are reconnected at points called nodes [17]. FEA has reformed scientific modeling in engineering methods and can also be used in the analysis of the components of the orofacial system [18,19,20,21,22]. 

The detection of non-carious abfraction lesions in the cervical area of dental hard tissues by the clinician is an alarm signal that can predict the patient’s need for occlusal balancing therapy in the future. The originality of this study consists in carrying out an experimental FEA study (which is a non-invasive repeatable method that does not require the presence of the patient, realized in accordance with data from the patient’s CBCT), and determining how the obtained results can represent a first step in the phasing of the dental treatment applied by the clinician to patients with abfraction lesions. Later, various occlusion analysis systems can be used to record detailed data regarding the existence of excessive occlusal forces (including the use of a T-Scan digital device), but these types of analyses can only be performed in the presence of the patient. The aim of the present study was to assess, using FEA, the behavior of hard dental tissues at the level of the lower premolar with abfraction under perpendicular and 45-degree inclined experimental forces.

## 2. Case Description

A 65-year-old female patient came to the dental practice for the treatment of dental pain. The patient complained of dental sensitivity to cold, hot, sweet, and sour agents in the lower right premolar area. Anamnesis revealed that the patient was not under treatment for chronic diseases. According to what was stated in the anamnesis, the patient had not previously undergone orthodontic treatments and had no traumatic injury. The patient also complained of the signs and symptoms of bruxism (teeth grinding or clenching loud enough to wake up the sleep partner, jaw and face soreness, tired or tight jaw muscles, and a dull headache in the temporal area). The patient declared that she did not drink fruit juices and other acidic drinks, that she has no digestive diseases (reflux and/or vomiting), that she uses a soft-bristled toothbrush and toothpaste containing fluoride, that her teeth are brushed gently, and that she does not wear a mouth guard or occlusal splint. 

During the extraoral examination, it was observed that the masseter muscles presented with hypertonicity, especially on the left side, and painful sensitivity in the temple area. 

**Figure 1:**
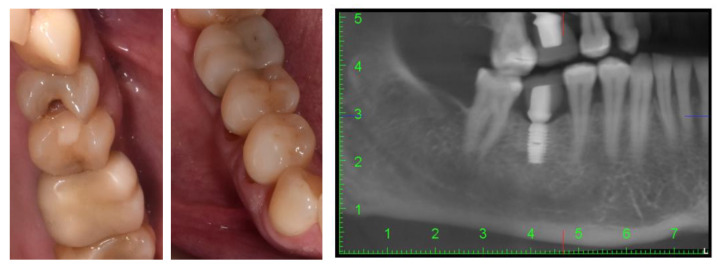
Intraoral aspects and CBCT image of patient.

The intraoral examination revealed that the patient presented with multiple simple odontal lesions that were partially treated, correctly and incorrectly. A simple carious lesion affected the right upper first premolar (1.4) (Figure 1). Wear areas were also observed in the incisal and occlusal regions of the right lower canine and premolars, as well as abfractions, with exposed dentine, located in the buccal cervical zone of the mandibular right premolars. The aspect of abfractions was mixed-shaped (wedge and saucer), located in enamel and dentine, without prepared cavities, and without any bacterial deposits. The abfraction lesions were 1.2 mm in depth. The most commonly used index that categorizes tooth wear in the cervical region is the Tooth Wear Index by Smith and Knight [23]. The abfraction lesions of the lower right premolars were determined to be 3rd class (defects ranging from 1 to 2 mm deep). 

The cervical margin of the patient’s abfraction lesions were located at the level of the free gingival margin. No gingival recessions were detected at the level of the cervical area of the two premolars. During the anamnesis and the clinical examination of the patient as well as later during the treatment and monitoring, no changes of periodontal tissues were detected (e.g., inflammation, gingival bleeding, the apparition of periodontal pockets, or dental mobility).

These mandibular right premolars presented with painful tooth sensitivity. The teeth appeal revealed the absence of 1.8, 1.6, 2.6, 3.5, 3.6, 4.6, and 4.8 teeth (after the FDI two-digit notation system). Cone-beam computed tomography (CBCT) of the orofacial area was indicated as a complementary examination. The patient’s missing teeth were already prosthodontically rehabilitated when she came to the dentist, through dental implants. She told us that these were short Bicon dental implants (facts confirmed by analysis of the CBCT images) with the superstructure made of hybrid ceramics. 

After the professional prophylactic cleaning of both dental arches, the patient was trained regarding the proper toothbrushing technique in order to abolish any eventual horizontal movements with excessive force during toothbrushing. 

The specific dental treatment started with the desensitization of abfraction areas (with GLUMA desensitizer, KulzerGmbH, Mitsui chemical group, Hanau, Germany) and the manufacturing of a thermoformed polyethylene occlusal mouth guard (of Sof-Tray Sheets, Ultradent Products Inc., South Jordan, UT, USA). The mouth guard had a thickness of 1.5 mm and was inserted on the lower dental arch. The patient was instructed on how to use the mouth guard to control the bruxism parafunction. 

## 3. Finite Element Analysis (FEA) of Mandibular Right Premolars

The data provided by the CBCT were also used for performing the finite element analysis (FEA). 

The FEM analysis in the presented case represented the first step in clarifying the stages of applied dental treatment in future sessions, especially since these experimental studies did not require the presence of the patient. By performing finite element analysis (FEA), it was possible to identify the harmful direction of forces as well as the actual and future structural deficiencies in the lower right premolar teeth with abfractions. 

To carry out the numerical analyses, we selected the clinical case of a 65-year-old patient who presented with abfractions at the level of the lower right premolar group. The patient underwent a CBCT at an imaging center, and the information provided by the computed tomography was processed with dedicated software and subsequently subjected to finite element analysis. The processing of the information provided by CBCT, and the numerical study, were carried out at the University POLITEHNICA of Bucharest. 

**Figure 2:**
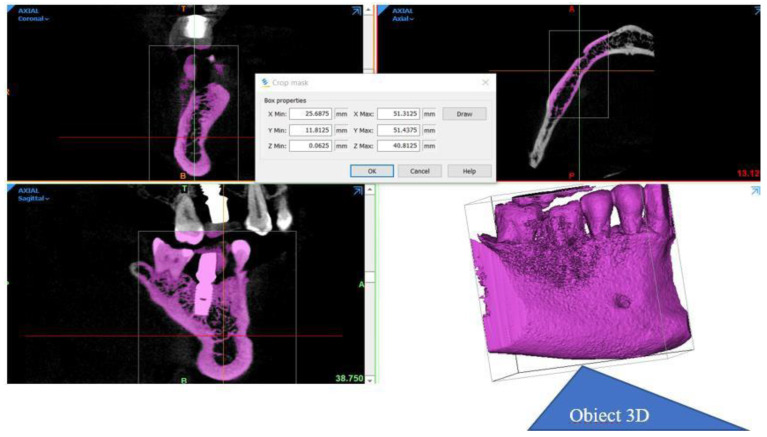
The stages in obtaining the desired anatomical area in 3D format.

Tomographic information was imported into the software package Mimics Innovation Suite 17, © Materialise NV. It has two components: Mimics and 3-matic. In Mimics, information from the CBCT was read in axial incidence whereupon the program immediately calculated the other two incidences, coronal and sagittal. The area of interest in the study was selected by applying the filters corresponding to predefined areas of Hounsfield units (grayscale masks). The mask was limited in its expansion by cropping. The desired anatomical area was created in a 3D format (Figure 2).

**Figure 3:**
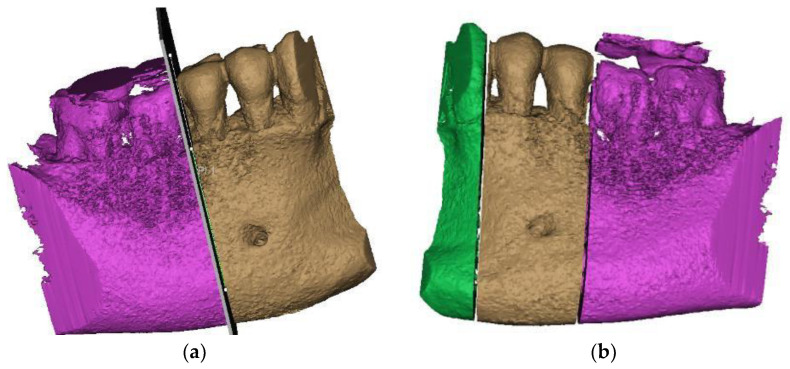
Sections to preserve the area of interest: (**a**) distal section; and (**b**) mesial section.

From the desired anatomical zone, the area of interest for the study was delimited through specific sectioning operations in order to be able to analyze the region of the two premolars affected by abfraction (Figure 3a,b).

**Figure 4:**
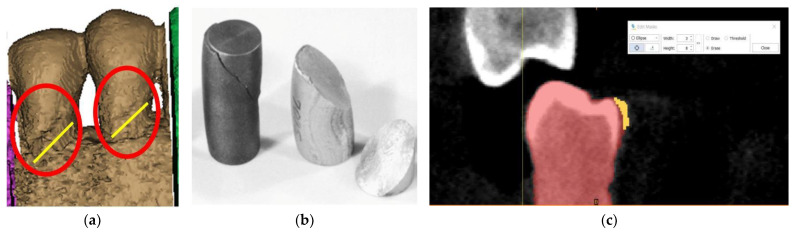
(**a**) Highlighting of abfraction areas; (**b**) compression fracture for cast iron and aluminum: “classic” fracture surface at 45°; and (**c**) selecting artifacts for removal.

By replicating the “crop” function, we managed to isolate the area of interest, where the presence of the abfraction at the bundle level is visible on the buccal faces of the two premolars (Figure 4a). The phenomenon is similar to the compression cracking produced in the case of cold plastic deformation of metallic materials (Figure 4b). The “editing” of the premolars is a laborious operation in the case of processing images obtained from CBCT. The Mimics program is being developed for studies based on images without artifacts. The elimination of artifacts (Figure 4c) was effectuated “pixel by pixel” on each slice of the tomography.

**Figure 5:**
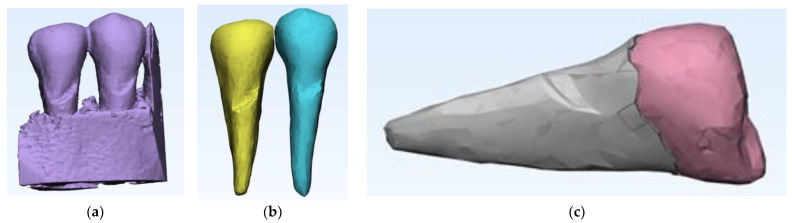
(**a**) Introducing data into 3-matic; (**b**) the steps for separating the anatomical components of interest in the 3-matrix; and (**c**) separation of cementum from enamel.

After the complete separation of the area of interest from possible artifacts, the data obtained with Mimics was further processed in 3-matic, the second component of the suite (Figure 5a). In 3-D, the anatomical components of interest were separated step by step so that, finally, the premolars were visualized as separate 3D objects (Figure 5b). To reach this stage, for each of the three objects (mandible, first and second premolar), we need to go through several stages of “repairing” their surfaces to be able to move from an external surface to 3D objects with volumetric consistency, which allows performing complex mechanical analyses with finite elements. It can be seen that the 2nd premolar presents a more extensive area of abfraction in surface and depth, which is why all calculations were subsequently performed only on this anatomical element. To perform a relevant analysis, cementum and enamel were separated in Mimics. This was done using density filtering of the original CBCT information (Hounsfield units), and then separate 3D objects were generated (Figure 5c).

**Figure 6:**
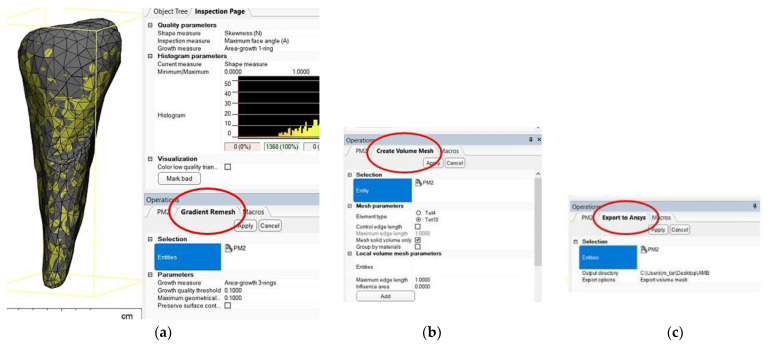
(**a**) Gradient remesh procedure; (**b**) creating the volumetric network; and (**c**) volumetric mesh export in ANSYS.

After the stage of obtaining an error-free 3D object, the recalculation of the triangulation mesh follows in order to move from the triangles that define the outer contour to tetrahedral elements through which the outer contour acquires an inner, volumetric “consistency”. The recalculation of the triangle mesh can be conducted in several ways, and it is even possible to reduce the number of tetrahedral elements to reduce the duration of the numerical calculation procedures. Figure 6a shows the situation after recalculation with a gradient mesh (procedure called gradient remesh). If the previous steps have gone correctly, the creation of the volumetric mesh can proceed (Figure 6b), and if the volumetric meshes has been calculated successfully, the anatomical objects processed in this way can be exported to numerical analysis programs with finite elements, in this case ANSYS © ANSYS Inc. (Houston, TX, USA) (Figure 6c). 

**Figure 7:**
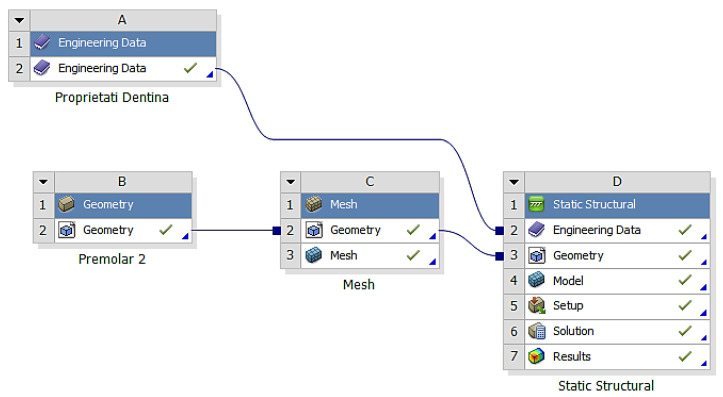
The structure of the module for calculating the effects of applied stress.

After importing the volumetric meshes into ANSYS, the structure of the module was selected for calculating the effects of the applied stress (Figure 7). 

**Figure 8:**
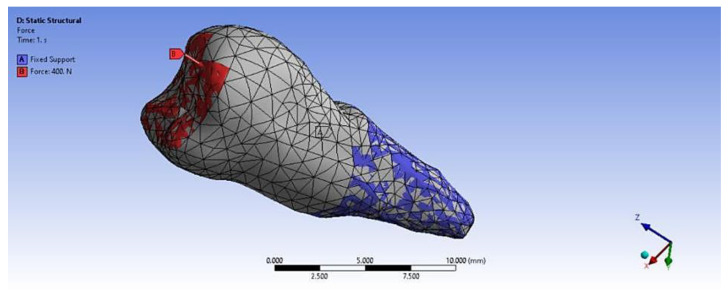
Definition of support area and force application areas.

In the analyzed case, we selected the Static Structural calculation mode, within which the objects that, through their geometry, constitute the model and the properties of the materials involved in the calculations (Engineering Data) were defined. Next, the definition of the Support (support of the volumetric element) and the means of applying mechanical stress (Force) were determined. In the Solution section, the mechanical elements that required calculation by FEA were determined. The adjacent diagram shows the requirements. Then, the program started to find results in the Solution section. In the case of the second premolar, the support area A inside the alveolus (blue area) and the area B for applying the pressure force to the occlusal surface (red areas) were defined (Figure 8).

**Figure 9:**
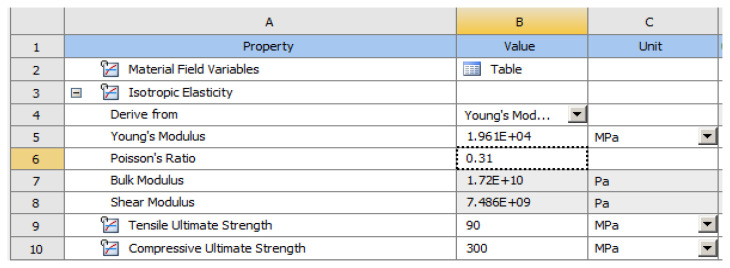
Material constants used in calculations.

In the Engineering Data (Figure 9), the material constants characterizing the tooth required for performing the analysis with finite elements were entered. A force of 400 N at 35 °C was used in two positioning variants: perpendicular to the tooth crown and inclined at 45°, to simulate mastication (the first variant) and stress due to bruxism (the second variant).

The elastic properties of cementum and enamel, entered into the software to perform the numerical simulations, are presented in Table 1.

Table 2 shows the characteristics of the two meshes (enamel and cement).

**Figure 10:**
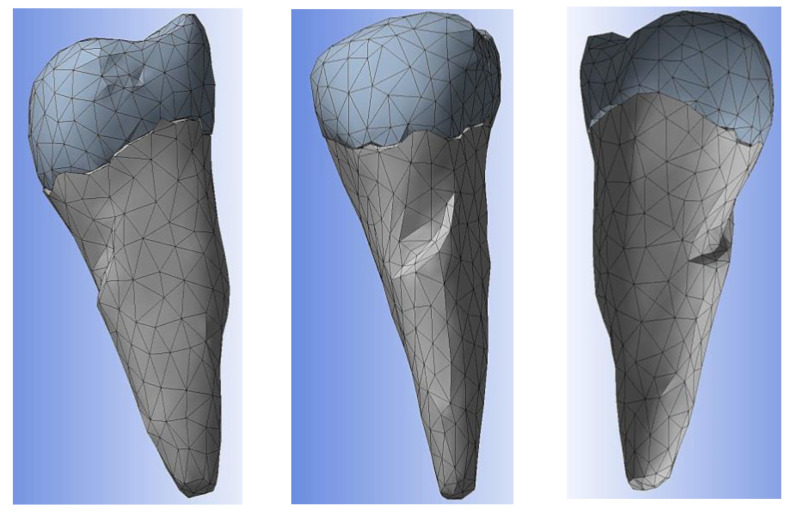
Enamel–cement assembly.

Figure 10 shows the enamel–cement assembly, the two layers that cover the crown and the root.

**Figure 11:**
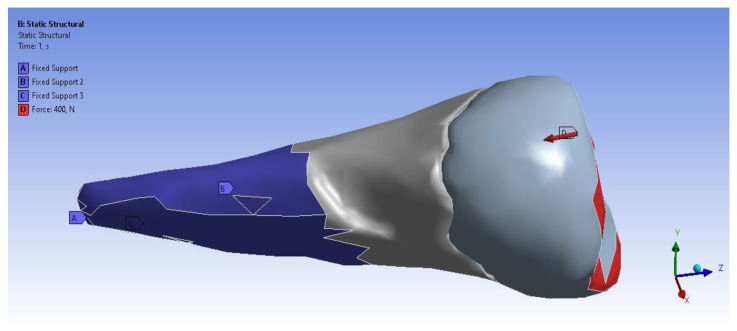
The force applied perpendicular to the occlusal surface and the support in the alveolus.

**Figure 12:**
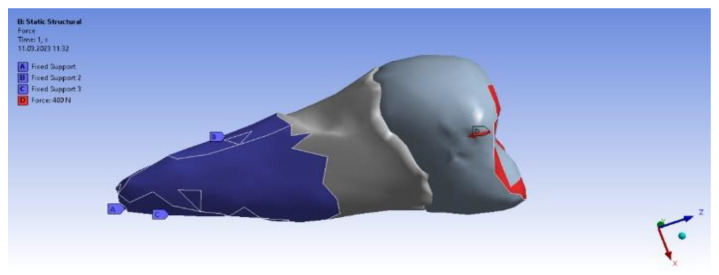
The force applied at an angle and the support in the alveolus.

Figure 11 shows the application of the experimental force perpendicular to the occlusal surface. Figure 12 presents the applying of the inclined force. In both figures, the root zone where the support in the alveolus was defined is marked in blue.

Table 3 present the distribution of the experimental forces applied perpendicularly and inclined at 45° on the 3 axes OX, OY, and OZ.

Table 4 and Table 5 show the centralized maximum and minimum values of the stresses and deformations produced as a result of the experimental solicitations applied perpendicularly and at a 45° inclination to the occlusal surface of the premolar.

**Figure 13:**
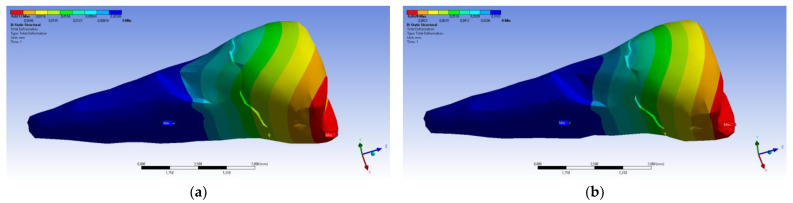
(**a**) The total deformation when the experimental force is applied perpendicular to the occlusal surface; and (**b**) the total deformation when the experimental force is applied at 45° on the occlusal surface.

The total deformation (Figure 13a,b) is apparently distributed similarly regardless of the direction of the experimental forces, except when the perpendicular forces are applied, when a value approximately three times than the effect produced after the inclined application of force is observed.

**Figure 14:**
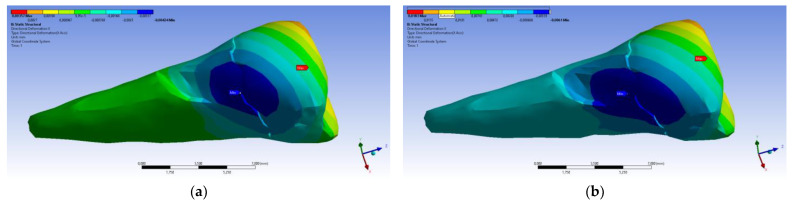
Deformation in the X direction when the experimental force is applied perpendicular to the occlusal surface: (**a**) mesio-buccal aspect; and (**b**) buccal aspect.

**Figure 15:**
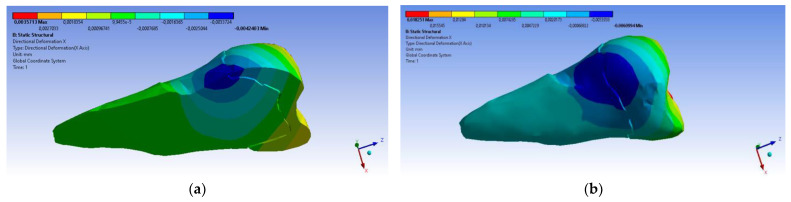

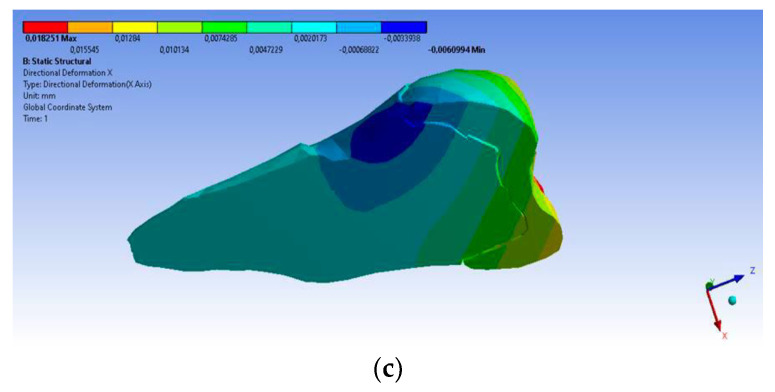
Deformation in the X direction when the experimental force is applied at 45° on the occlusal surface: (**a**) bucco-lingual section; (**b**) mesio-buccal aspect; and (**c**) mesio-buccal section.

A small contraction (~−2 × 10^−3^ mm) was recorded in the X direction (Figure 14 and Figure 15) in the area bordering the abfraction. If the greatest contraction is manifested buccally at the enamel–cementum junction and lingually in the coronal region, a low elongation occurs (~3 × 10^−4^ mm).

**Figure 16:**
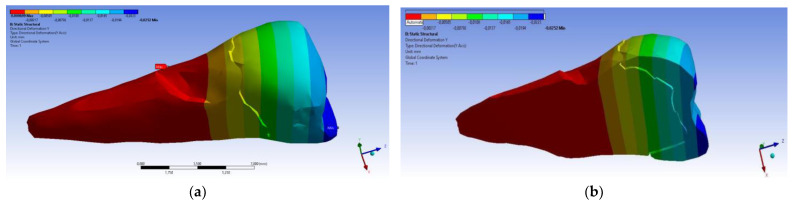
Deformation in the Y direction when a perpendicular experimental force is applied to the occlusal surface: (**a**) mesio-buccal aspect; and (**b**) section.

**Figure 17:**
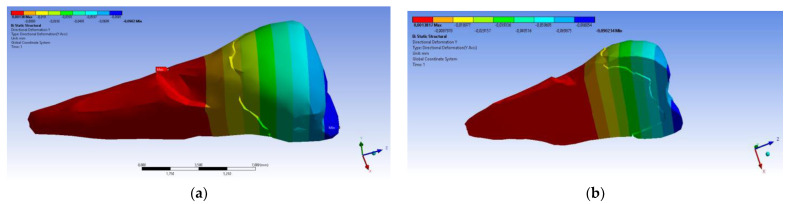
Deformation in the Y direction when a 45° experimental force is applied on the occlusal surface: (**a**) mesio-buccal aspect; and (**b**) section.

As for the deformation in the Y direction, the behavior was similar (Figure 16 and Figure 17), but it was found that the inclination of the force causes 3–4 times larger deformations.

**Figure 18:**
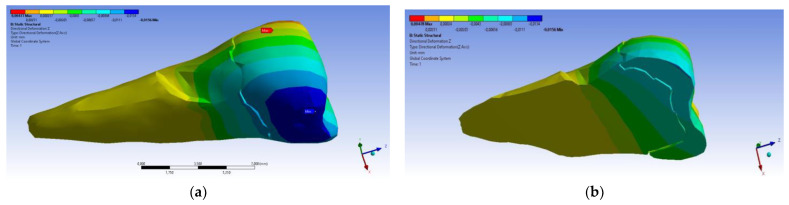
Deformation in the Z direction when the experimental force is applied perpendicular to the occlusal surface: (**a**) mesio-buccal aspect; and (**b**) section.

**Figure 19:**
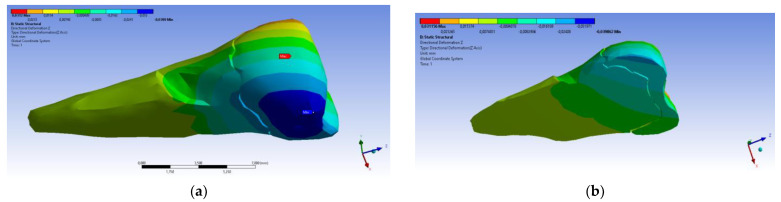
Deformation in the Z direction when the 45° experimental force is applied on the occlusal surface: (**a**) mesio-buccal aspect; and (**b**) section.

In the Z direction (Figure 18 and Figure 19), the deformation was also greater in the case of a 45° angle in the experimental force direction on the occlusal surface.

**Figure 20:**
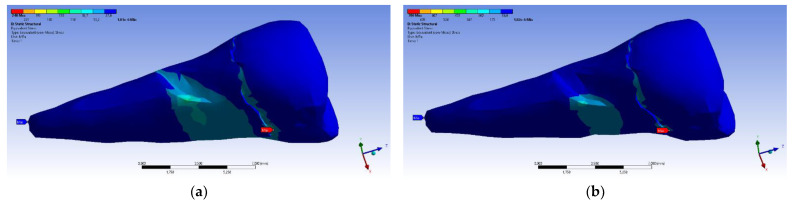
Equivalent stress when the experimental force is applied: (**a**) perpendicular to the occlusal surface (mesio-buccal aspect); and (**b**) at 45° on the occlusal surface (section).

In the case of perpendicular force application, the affected area of the abfraction lesion manifested relatively moderate values of the equivalent stress (80 MPa) (visible in Figure 20a). Higher maximum stresses (~3 times) developed in the case of inclined force application (Figure 20b), but it can be seen that the stress transmission was only superficial.

**Figure 21:**
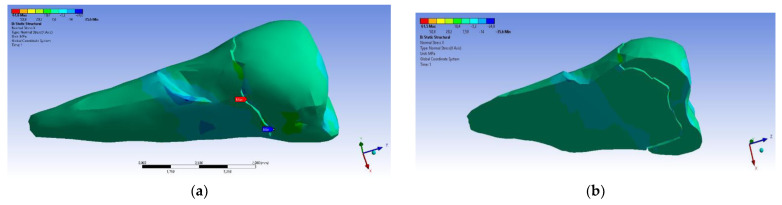
Localizations of normal stress X when a perpendicular experimental force is applied on the occlusal surface: (**a**) mesio-buccal aspect; and (**b**) section.

**Figure 22:**
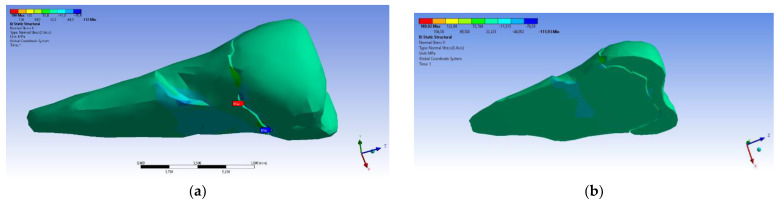
Localizations of normal stress X when an experimental force at 45° is applied on the occlusal surface: (**a**) mesio-buccal aspect; and (**b**) section.

On the lower flank of the abfraction area, a normal compressive stress on the X axis appears, with a value of −35.6 MPa, visible in Figure 21a,b. An almost similar situation, but with stresses that were three times higher, occurred in the case of the inclined force application (Figure 22a,b). 

**Figure 23:**
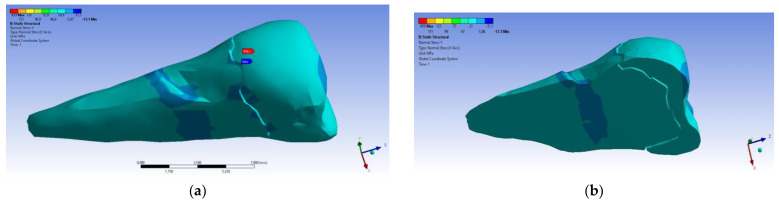
Localizations of normal stress Y when a perpendicular experimental force is applied on the occlusal surface: (**a**) mesio-buccal aspect; and (**b**) section.

**Figure 24:**
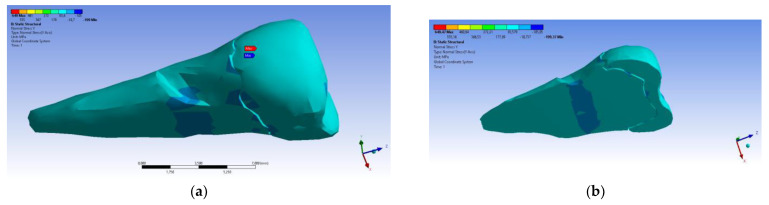
Localizations of normal stress Y when an experimental force is applied at 45° on the occlusal surface: (**a**) mesio-buccal aspect; and (**b**) section.

In the Y direction, relatively high compressive stresses (~−20 MPa) appeared in the parcel area, which also propagated to the section of the affected area. The recorded values were 3–4 times higher when the force was applied in an inclined direction (Figure 23 and Figure 24).

**Figure 25:**
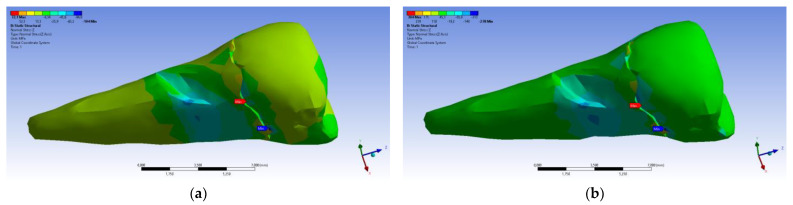
Localizations of normal stress Z when experimental forces are applied to the occlusal surface: (**a**) perpendicular forces (mesio-buccal aspect); and (**b**) a 45° force is applied to the occlusal surface (aspect).

In the Z direction, the same tendency was maintained (Figure 25).

**Figure 26:**
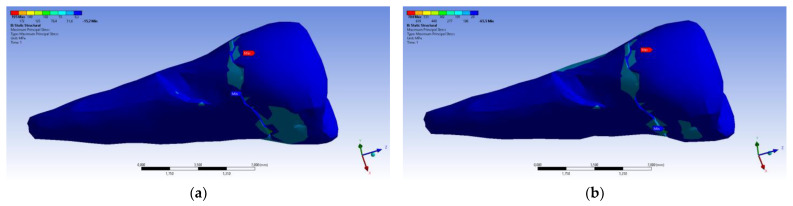
Mesio-buccal aspect of maximum principal stress when experimental force is applied to the occlusal surface: (**a**) perpendicularly; and (**b**) at 45°.

The maximum principal stress registered maximum values in the package area, both when the experimental force was applied perpendicular to the occlusal surface (Figure 26a) and when it was applied at an angle of 45° (Figure 26b).

**Figure 27:**
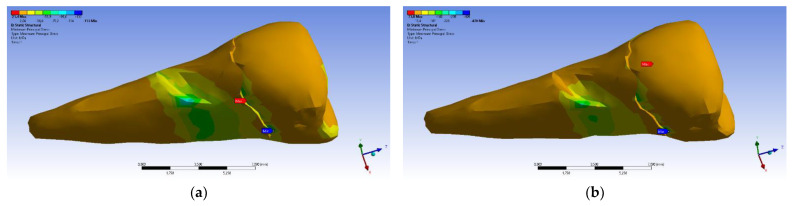
Maximum principal stress when experimental forces are applied to the occlusal surface: (**a**) perpendicularly; and (**b**) at 45°.

If, in the apical 2/3 of the root and in the coronal area of the tooth, the minimum main stresses were tensile around the abfraction zone, when the applied experimental force was perpendicular to the occlusal surface, in the cervical 1/3 of the root, these stresses changed and became compressive, as depicted in Figure 27a. In accordance with previous observations, the behavior was similar when a 45° experimental force was applied to the occlusal surface, like in Figure 27b. 

**Figure 28:**
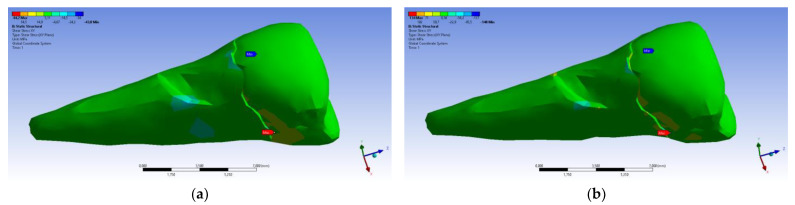
Mesio-buccal aspect of tangential tension XY when the experimental force is applied to the occlusal surface: (**a**) perpendicularly; and (**b**) at 45°.

**Figure 29:**
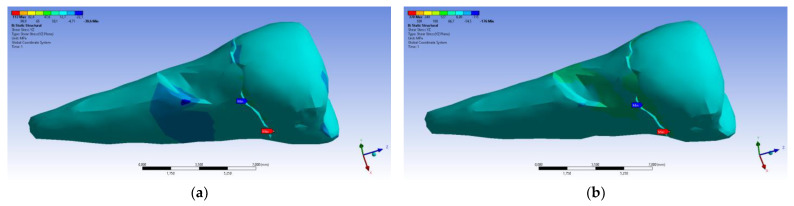
Mesio-buccal aspect of tangential tension YZ when the experimental force is applied to the occlusal surface: (**a**) perpendicularly; (**b**) at 45°.

The tangential stresses in the XY, YZ, and XZ directions were manifested in the same way on the higher values, corresponding to the inclined application of the forces (Figure 28 and Figure 29). 

**Figure 30:**
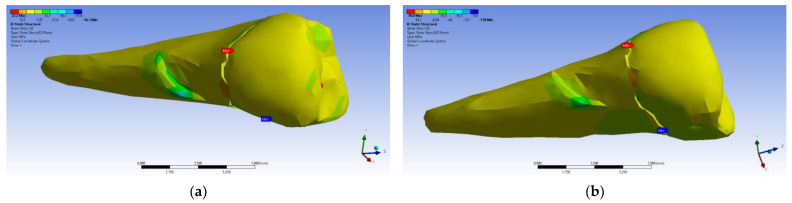
Mesio-buccal aspect of tangential tension XZ when the experimental force is applied to the occlusal surface: (**a**) perpendicularly; and (**b**) at 45°.

The tangential stresses in the YZ plane were compressive (~−20 MPa), while the rest of the tooth was predominantly subjected to slightly higher but tensile stresses. Moderate compressive stresses were manifested in the XZ plane, but also precisely in the affected area (Figure 30).

**Figure 31:**
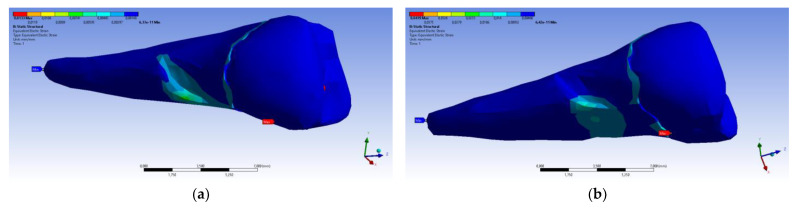
Mesio-buccal aspects of the maximum shear elastic strain when applying the experimental force to the occlusal surface: (**a**) perpendicularly; (**b**) in 45°.

Figure 31 presents the mesio-buccal aspects of the maximum tangential stress when the experimental force was applied to the occlusal surface perpendicularly (a) and at 45° (b). The developed maximum tangential stress was tensile, and in the affected area it had a moderate value.

**Figure 32:**
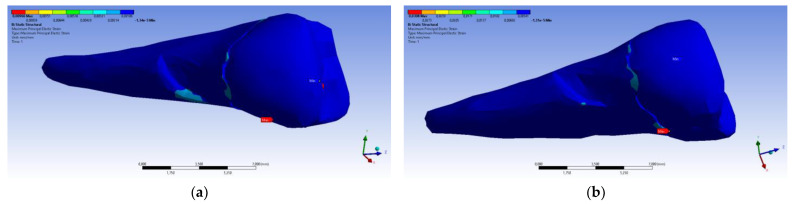
Mesio-buccal aspects of the maximum principal elastic strain when applying the experimental force to the occlusal surface: (**a**) perpendicularly; and (**b**) at 45°.

**Figure 33:**
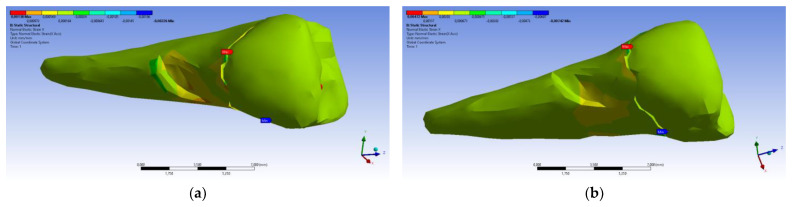
Mesio-buccal aspects of the normal elastic strain X when applying the experimental force to the occlusal surface: (**a**) perpendicularly; and (**b**) at 45°.

**Figure 34:**
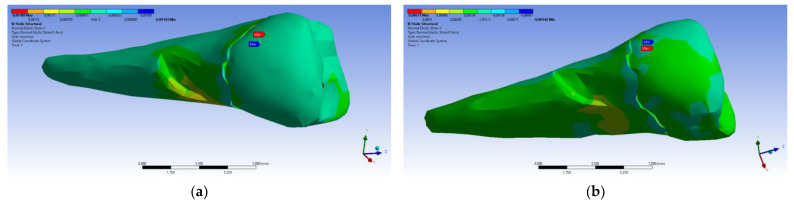
Mesio-buccal aspects of the normal elastic strain Y when applying the experimental force to the occlusal surface: (**a**) perpendicularly; and (**b**) at 45°.

**Figure 35:**
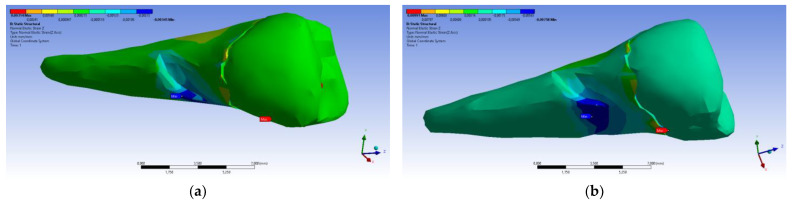
Mesio-buccal aspects of the normal elastic strain Z when applying the experimental force to the occlusal surface: (**a**) perpendicularly; and (**b**) at 45°.

**Figure 36:**
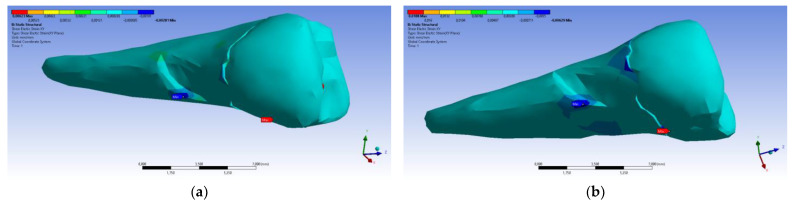
Mesio-buccal aspects of the shear elastic strain XY after the application of an experimental force to the occlusal surface: (**a**) perpendicularly; and (**b**) at 45°.

**Figure 37:**
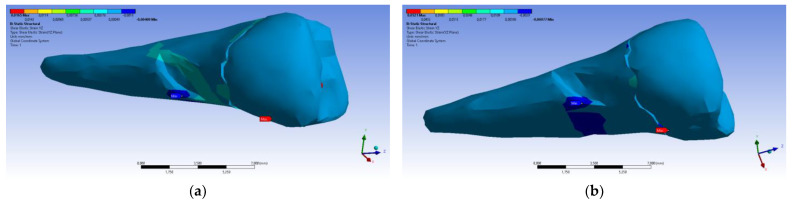
Mesio-buccal aspects of the shear elastic strain YZ after the application of an experimental force to the occlusal surface: (**a**) perpendicularly; and (**b**) at 45°.

**Figure 38:**
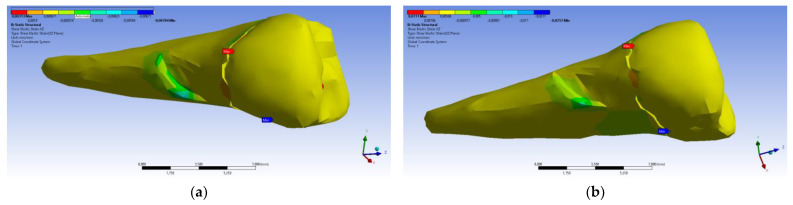
Mesio-buccal aspects of the shear elastic strain XZ after the application of an experimental force to the occlusal surface: (**a**) perpendicularly; and (**b**) at 45°.

When determining elastic relative deformation (Figure 32a,b), normal elastic relative deformation X (Figure 33a,b), Y (Figure 34a,b), Z (Figure 35a,b), the relative tangential elastic deformation XY (Figure 36a,b), YZ (Figure 37a,b) and XZ (Figure 38a,b), the cervical third of the root around the abfraction area always behaves differently than the other areas of the tooth. 

As in the case of the other determinations, the inclined application of the forces will lead to the development of more important deformations than in the case of the perpendicular application of the occlusal stresses. 

The experimental FEA research of the forces exerted on the lower premolars with abfraction lesions was carried out in order to demonstrate the fact that the axial over-stressing forces and those applied at an angle of 45 degrees possibly induce the appearance of unwanted effects on the dental hard tissues in their cervical areas.

The patient was assessed during further dental visits every month for 3 months. She continued to complain about the signs of bruxism, but without experiencing the painful sensitivity in the lower right premolars with abfraction. For this reason, it was indicated that the patient should continue wearing the custom-made mouth guard. 

After 8 months of wearing the customized thermoformed mouth guard, the patient agreed to participate in the recording of dynamic occlusal measurements using a digital T-Scan device. This device determined the levels of occlusal loads on the teeth of both dental arches, especially on the lower mandibular premolars that had abfraction lesions. In this way, the subsequent dental therapy necessary to obtain a stable occlusion of the patient’s teeth could be established.

## 4. Literature Review 

To accomplish this systematic literature review, a bibliographic survey was carried out in national and international scientific journals indexed in medical databases such as EBSCO, Medline, Google Scholar, PubMed, SciELO, Scopus, Web of Science. The keywords included “noncarious cervical lesion (NCCL)”, “abfraction”, “premolars”, “finite element method (FEM)”, and “finite element analysis (FEA)” in the title or abstract. 

These searches included English-language articles published between January 2014 and December 2023, tracking original research with full access. All identified articles were chosen according to their relevance and objectivity in relation to the topic under study. Data collection involved the study’s selection and data extraction. The article selection for review was undertaken separately by two reviewers towards their possible inclusion after their eligibility criteria were determined. In the first phase, 198 articles and abstracts were chosen. 

The inclusion criteria were as follows: the presence of the keywords applied for searching, articles with the search terms in the title or abstract, and original articles published between the years 2014 and 2023. 

The exclusion criteria were as follows: non-English language specialty literature; case reports; abstracts; editorial letters; laboratory and animal studies; studies based on questionnaires; treatment management studies; literature reviews, citations, patents, books, dissertation theses, reference books, print-ahead articles, articles that were outside the theme researched for the review, and published before 2014 or after 2023. 

Thirty-four articles were eliminated as not related to the research topic. The remaining 29 articles were screened to exclude duplicates and irrelevant papers. Eighteen articles published before 2014 or after 2023 were discarded after reading and analysis according to the exclusion criteria.

In the next stage, references in the included articles were also read in order to identify additional relevant literature. Three researchers extracted the data, and the other three validated it. The eligibility of the articles included in the review was resolved by consensus, with no disagreements. After applying all the selection criteria (derived from the inclusion and exclusion criteria), only eleven articles remained in the study for this review.

A brief description of the included articles in this study is presented in Table 6, and the results and conclusions related to those articles are shown in Table 7 [24,25,26,27,28,29,30,31,32,33,34].

## 5. Discussion

Dental enamel, the densest calcified tissue in the body, contains, by weight, 96% inorganic material (represented by hydroxyapatite, HA), 1–2% organic material (proteins), and water. [35,36,37]. HA crystal orientation confers the notable mechanical properties of enamel [38]. The cement–enamel junction (CEJ) is a very sensitive zone in the cervical area of teeth, and the physiologic biomechanics of enamel and CEJ functions are difficult to study [39,40]. In the lateral areas of dental arches with interferences, the existence of parafunctional forces can influence the normal morphology of the CEJ. The amount of the organic phase at the interface influences the deformation of dental hard tissues at the enamel–dentine junction. The repeated parafunctional forces can generate excessive compressive or shearing pressure, which induces the apparition of microcracks in the HA crystals. The crack propagation in the enamel is less due to its rigid structure, and most cracks appear near the external coat of the enamel. In time, the microcracks can extend until the enamel and dentin are degraded and determine the deformation of that area [41,42,43]. The deformation differs at the enamel–dentine (EDJ) and cement–dentine (CDJ) junctions. The propagation of cracks is due to the rigid structure of the external layer of enamel, where the majority of microcracks are located [43,44]. The ability to maintain, through fluid percolation, the crystalline integrity in the hydroxyapatite of enamel and dentin under occlusal loads is limited [37,43]. Exposure to repetitive mechanical loads results in cumulative fatigue damage. The dysfunctional and excessive action of masticatory forces applied non-axially to malpositioned or bruxing teeth causes flexion of the teeth’s crystalline structure, which induces destruction of the prismatic architecture of HA and the apparition of the specific shape of abfraction lesions due to the low packing density of the Hunter–Schreger band (HSB) in the cervical area [8,10,11]. The highest occurrence of abfractions is found in the mandibular premolars, followed by the maxillary premolars, and then by the molars, canines, and incisor teeth. This fact is probably induced by the presence of the furcation in the area adjacent to the cervical coronal area, the cervical constriction of the crown, and its small volume [7,8,10]. Chewing cycle loads can cause significant bending and flexural loads on the cusps of posterior teeth in the time of functional and parafunctional occlusal movements [45,46]. These occlusal loads can theoretically cause the occurrence of the horizontal force component too, which can determine the accumulation of tensile stress and further, the apparition of cracks in the inter-crystalline space [46,47]. 

The knowledge of the etiological factors in abfraction lesions and in all types of NCCLs is of major significance in order to conduct a proper diagnosis and, therefore, to realize accurate therapeutic management of NCCLs. The utilization of virtual investigations, like finite element analysis (FEA), represents an actual tendency in dentistry. Through FEA, it is possible to examine the stresses and strains of intricate systems like the orofacial system. This involves the mathematical conversion and analysis of the mechanical parameters of an object [19,21,22,35]. FEA allows for the simulation of possible clinical situations that may be encountered in practice in order to estimate the behavior of some dental structures under conditions of maximum stress [21,33,48]. 

Abfraction lesions represent a demineralization of the human body and can occur in association with pathological wear of the tooth due to the interaction of chemical, biological, and behavioral factors [36]. The biomechanics of abfraction lesions can also be indirectly evaluated by using the FEA method [10,19]. In our FEA study, 400 N extrinsic forces were applied perpendicularly and tilted at 45° on three axes, XY, OY, and OZ, which were high enough to induce the breakdown of cement and dentine HA crystals.

Tanaka et al. [49] realized an analysis of stress on a maxillary central incisor and mandibular first molar by using the two-dimensional finite element method (FEM) and applying the elasto–plastic deformation theory. They observed that the oblique loading spreads on the enamel surface near the CEJ and provokes plastic distortions, which in time can lead to the apparition of NCCLs. Stănuși et al. [31] observed that the values and distribution of strain were not favorable in the application of excessive horizontal loads on the studied undamaged tooth. They also pointed out that the tooth that was undamaged was the most affected by stress, regardless of the force used. In another FEA study with occlusal loads in a maxillary first premolar, the zones with an excessive concentration of forces were highlighted. The authors observed that in all models (with horizontal or oblique wear on intact tooth), stress was situated especially in the cervical area of the buccal surface of the tooth, and that the most harmful effects were caused by the excessive intensity of applied forces [33]. In conformity with the results of our FEA study, the inclined application induced stresses and deformations that were three to four times higher than in the case of the perpendicular application of experimental forces.

The dental cracks can be understood as predecessors to the formation of NCCLs, and many researchers have reported an interrelationship between occlusal loads and stress and the development of NCCLs [12,30,33,48,50,51,52]. Research by Donovan et al. [53] indicated that excessive vertical occlusal loads are associated with the progression of all types of NCCLs. The results of our study are in accordance with their findings, and the investigations also showed that the inclined application of the forces will lead to the development of more important deformations than in the case of a perpendicular application of occlusal stresses. Our study revealed, also, that there is a correlation between perpendicular and 45°-inclined occlusal loads and the occurrence of stress in the buccal-cervical area of the tooth. 

After the research conducted by Poiate et al. [54], FEA has become appropriate for quantitative stress analysis and provides important results related to stress and displacement that are fundamental to planning preventive and restorative approaches in abfractions. Vuković A. et al. [32] used the FEA method to evaluate the distribution of stress and deformation on the lower first premolar by applying 200 N axial and non-axial loads. The stress values situated in the cervical area of the intact tooth were higher in the region of the EDJ, and the deformation values of the tooth under para-axial loads were approximately 10 times higher than the value of the deformation under an axial load. 

The FEA study performed by Yang et al. [55] indicated that the developed stress appeared in axial and non-axial occlusal charges according to the level of periodontal bone support, which was induced by the hard and soft tissues of a mandibular premolar. The applied stress of 90 N was on the same buccal cusp occlusal slope, both axially and non-axially (at 45°). The stress in both the radicular dentine and the periodontal bone ridge was unevenly distributed due to the non-axial forces that were used. There was a significant increase in cases of severe bone reduction (≥50%). The authors also found that the decrease in periodontal bone support can induce the apical expansion of the tooth defect. Our analysis reported that the occlusal stresses’ perpendicular solicitations lead to less significant deformations than in the case of the inclined application of the forces.

Excessive loads exerted on teeth can induce the development of periodontal disorders. In their research, Luchian et al. studied, through FEM, the occurrence of periodontal damage to mandibular incisors under the action of different degrees of orthodontic forces and the potential risks for the development of periodontal diseases, although these effects were still not clinically recognized. They concluded that the anatomical particularities should be taken into consideration in the application of orthodontic forces to incisors with damaged periodontal tissues [56].

Many authors consider that, although time-consuming, FEA analysis helps us to explain the occurrence of a series of unwanted phenomena at the level of some teeth, implants, direct or indirect restorations, and the dental materials used, and can indicate the vulnerable areas where maximum stresses and deformations are developed [57,58,59,60,61,62]. Jakupović A. et al. [63] found that the type of tooth loading has the greatest influence on the intensity of stress. The values of the obtained stresses in the restorative material and dental tissues differ due to the different mechanical properties of the materials. Restoration of NCCLs significantly reduces extremely high stress values at their bottom. Dam Van et al. [64] underlined that FEA is an important tool in implant dentistry to study the stress distribution on the adjoining bone, the biomechanics of dental implants and bone, the implant and bone interface, and their fatigue behaviors. Other authors concluded that since the experimental capabilities in implant dentistry are greatly limited by the ethical aspects of research on human subjects, FEAs are useful procedures for understanding the micro-displacements of dental implants and the mode of transmission of stresses, strains, and deformations at the bone level, with direct implications for the involved tissues [19,65,66,67,68,69,70].

Finite element analysis is appropriate for quantitative static stress analysis and provides important results for both stress and displacement. These are fundamental to planning preventive and restorative approaches in NCCLs [69,70,71]. Although this FEA study has useful results for establishing correlations with clinical data, it still has limitations, which should be considered. 

The controversial etiology and questionable diagnosis of NCCLs have often led to an erroneous approach to their therapeutic management. Through FEA results, the stress distribution values of dental hard tissues, which are appreciably impacted by their properties, like the elastic modulus and surface properties, can be highlighted [72]. FEA also enables the evaluation of the fracture risk of a tooth or dental implant in accordance with the distribution and orientation of stress [62,64]. 

Our study is limited in that it examined a single case. Longitudinal studies in enlarged samples will make it possible to assess the development of abfraction lesions due to occlusal loads and interferences and test the efficiency of dental materials and restorative treatments. Another limitation of the study is that FEA is a computerized investigation realized after patient data were input from CBCT, which means that the clinical conditions may not have been totally reproduced. FEA studies should always be completed with a personalized clinical evaluation. The exactitude and the material properties of the data utilized in the model influence the FEA predictions [19]. Currently, the involvement of occlusal forces in the formation of structural defects in abfractions is beyond question. 

The detection of non-carious abfraction lesions in the cervical area of the dental hard tissues by the clinician (especially at the level of the upper canines and the maxillary and mandibular premolars) indicate that the patient will require occlusal balancing therapy in the future. Various ulterior occlusion analysis systems can be used to record detailed data regarding the existence of excessive occlusal forces (including the T-Scan digital device), but these types of analyses can only be performed in the presence of the patient.

This study recognized the relationship between the morphology of the studied teeth and the effect of axial and inclined forces on the apparition and advancement of NCCLs. The clinical relevance of the study is that it reinforces the use of FEA to realize scientific validation of the biomechanical features regarding hard dental structures, their supporting tissues, and the dental biomaterials used in direct and indirect oral rehabilitation. 

The results of this study may not be postulated to the general population because the cause of abfraction lesions is multifactorial and, at the same time, each patient presents individual characteristics that conventionally cannot be generalized.

The implications of this study for current dental practice should be emphasized. When examining a patient for the first time, the presence of abfraction lesions located in the cervical area of the teeth can indicate, to the clinician, possible overloading of the occlusal forces, which is why the clinician must perform a detailed history of the disease, a thorough anamnesis, and a complete examination of the patient. Future applications should focus on strategies for decreasing or eliminating paraxial loads in the optimization of temporo-mandibular joint models, teeth, dental implants, dentures, orthodontic appliances, etc., for proper dental therapeutic management.

The diagnosis and correction of occlusal imbalances are easier to achieve with inter-disciplinary collaboration. In this manner, the choice of treatment should be realized in accordance with other clinical and complementary examinations in order to increase the patient’s quality of life, because oral health is a major critical factor for general health and wellbeing.

## 6. Conclusions

The realized FEA demonstrated that the most vulnerable areas, where shears, stresses, and maximum deformations occurred, were in the cervical areas of the abfraction lesions.

The stresses occurred regardless of the ways in which the force was applied (perpendicular or inclined at 45°). In all cases, the inclined application led to a three-fourths higher development of stresses and deformations than in the case of the perpendicular application of the experimental forces.

Using computer simulations in imaging analysis and mechanical efforts through FEA can provide valuable information and offer solutions for a better understanding of the mechanisms involved in the masticatory stresses and strains on teeth.

## Figures and Tables

**Table 1:** Elastic properties of dental cement and enamel.

	Elastic Properties of Cement	Elastic Properties of Enamel
Young’s modulus, MPa	18,600	84,000
Poisson’s ratio	0.31	0.3
Global Modulus, MPa	16.316	70.000
Transverse Modulus, MPa	7099.2	32.308

**Table 2:** Characteristics of dental components.

Material	Condition	Statistics	
		Nodes	Elements
Cement	Built mesh	2685	1402
Enamel		2578	1316

**Table 3:** The distribution of the applied experimental perpendicular load and 45° load.

Experimental Load Applied Perpendicularly		Experimental Load Applied Inclined at 45°	
Type	Load	Type	Load
X component	−78 N (slope)	X component	−55 N (slope)
Y component	−78 N (slope)	Y component	−253 N (slope)
Z component	−385 N (slope)	Z component	−305 N (slope)
Resultant	400 N	Resultant	400 N

**Table 4:** Results obtained from the solicitation applied perpendicular to the occlusal surface.

	Total Deformation	Deformation in X Direction	Deformation in Y Direction	Deformation in Z Direction	Equivalent Stress	Normal Stress X	Normal Stress Y	Normal Stress Z	Maximum Main Stress	Minimum Main Stress	Tangential Stress XY	Tangential Stress YZ	Tangential Stress XZ
**Minimum**	0 mm	−4.24 × 10^−3^ mm	−2.52 × 10^−2^ mm	−1.56 × 10^−2^ mm	1.01 × 10^−6^ MPa	−35.6 MPa	−57.1 MPa	−104 MPa	−15.2 MPa	−152 MPa	−43.7 MPa	−39.6 MPa	−56.3 MPa
**Maximum**	2.77 × 10^−2^ mm	3.57 × 10^−3^ mm	6.98 × 10^−4^ mm	4.78 × 10^−3^ mm	248 MPa	61.5 MPa	177 MPa	72.2 MPa	195 MPa	21.4 MPa	44.2 MPa	117 MPa	22.2 MPa
**Minim. in**	Cementum	Cementum	Enamel	Enamel	Cementum	Cementum	Enamel	Enamel	Enamel	Cementum	Enamel	Enamel	Cementum
**Maxim. in**	Enamel	Enamel	Cementum	Enamel	Cementum	Enamel	Enamel	Enamel	Enamel	Enamel	Cementum	Cementum	Cementum
	**Maximum Tangential Stress**	**Main Elastic Relative Deformation**	**Main Maximum Elastic Relative Deformation**	**Main Minimum Elastic Relative Deformation**	**Maximum Tangential Elastic Relative Deformation**	**Relative elastic Normal X Deformation**	**Relative Elastic Normal Y Deformation**	**Relative Elastic Normal Z Deformation**	**Relative Elastic Tangential XY Deformation**	**Relative Elastic Tangential YZ Deformation**	**Relative Elastic Tangential XZ Deformation**		
**Minimum**	5.4 × 10^−7^ MPa	6.36 × 10^−11^ mm/mm	−1.33 × 10^−5^ mm/mm	−1.05 × 10^−2^ mm/mm	7.6 × 10^−11^ mm/mm	−2.26 × 10^−3^ mm/mm	−1.43 × 10^−3^ mm/mm	−3.45 × 10^−3^ mm/mm	−2.81 × 10^−3^ mm/mm	−4.09 × 10^−3^ mm/mm	−7.93 × 10^−3^ mm/mm		
**Maximum**	143 MPa	1.33 × 10^−2^ mm/mm	9.66 × 10^−3^ mm/mm	1.09 × 10^−5^ mm/mm	2.01 × 10^−2^ mm/mm	1.37 × 10^−3^ mm/mm	1.89 × 10^−3^ mm/mm	3.14 × 10^−3^ mm/mm	6.23 × 10^−3^ mm/mm	1.65 × 10^−2^ mm/mm	3.13 × 10^−3^ mm/mm		
**Minim. in**	Cementum	Cementum	Enamel	Cementum	Cementum	Cementum	Cementum	Cementum	Cementum	Cementum	Cementum		
**Maxim. in**	Cementum	Cementum	Cementum	Cementum	Cementum	Cementum	Cementum	Enamel	Cementum	Cementum	Cementum		

**Table 5:** Results obtained from the solicitation applied at 45° to the occlusal surface.

	Total Deformation	Deformation in X Direction	Deformation in Y Direction	Deformation in Z Direction	Equivalent Stress	Normal Stress X	Normal Stress Y	Normal Stress Z	Maximum Main Stress	Minimum Main Stress	Tangential Stress XY	Tangential Stress YZ	Tangential Stress XZ
**Minimum**	0 mm	−6.1 × 10^−3^ mm	−9.02 × 10^−2^ mm	−3.99 × 10^−2^ mm	1.02 × 10^−6^ MPa	−112 MPa	−199 MPa	−278 MPa	−65.5 MPa	−470 MPa	−148 MPa	−176 MPa	−178 MPa
**Maximum**	9.28 × 10^−2^ mm	1.83 × 10^−2^ mm	1.38 × 10^−3^ mm	3.12 × 10^−2^ mm	780 MPa	61.5 MPa	649 MPa	304 MPa	704 MPa	73.8 MPa	134 MPa	370 MPa	78.6 MPa
**Minim. in**	Cementum	Cementum	Enamel	Enamel	Cementum	Cementum	Enamel	Enamel	Enamel	Cementum	Enamel	Enamel	Cementum
**Maxim. in**	Enamel	Enamel	Cementum	Enamel	Cementum	Enamel	Enamel	Enamel	Enamel	Enamel	Cementum	Cementum	Cementum
	**Maximum Tangential Stress**	**Main Elastic Relative Deformation**	**Maximum Main Elastic Relative Deformation**	**Minimum Main Elastic Relative Deformation**	**Maximum Tangential Elastic Relative Deformation**	**Relative Elastic in Normal X Deformation**	**Relative Elastic in Normal Y Defomation**	**Relative Elastic in Normal Z Deformation**	**Relative Elastic Tangential XY Deformation**	**Relative Elastic Tangential YZ Deformation**	**Relative Elastic Tangential XZ Deformation**		
**Minimum**	5.45 × 10^−7^ MPa	6.42 × 10^−11^ mm/mm	−1.31 × 10^−5^ mm/mm	−3.26 × 10^−2^ mm/mm	7.68 × 10^−11^ mm/mm	−7.42 × 10^−3^ mm/mm	−5.42 × 10^−3^ mm/mm	−7.58 × 10^−3^ mm/mm	−6.29 × 10^−3^ mm/mm	−9.77 × 10^−3^ mm/mm	−2.51 × 10^−2^ mm/mm		
**Maximum**	450 MPa	4.19 × 10^−2^ mm/mm	3.08 × 10^−2^ mm/mm	7.02 × 10^−5^ mm/mm	6.34 × 10^−2^ mm/mm	4.72 × 10^−3^ mm/mm	6.75 × 10^−3^ mm/mm	9.91 × 10^−3^ mm/mm	1.88 × 10^−2^ mm/mm	5.21 × 10^−2^ mm/mm	1.11 × 10^−2^ mm/mm		
**Minim. in**	Cementum	Cementum	Enamel	Cementum	Cementum	Cementum	Cementum	Cementum	Cementum	Cementum	Cementum		
**Maxim. in**	Cementum	Cementum	Cementum	Cementum	Cementum	Cementum	Cementum	Enamel	Cementum	Cementum	Cementum		

**Table 6:** Brief description of included articles in the study [24,25,26,27,28,29,30,31,32,33,34].

Author, Reference No., Year	Title of Article	Aim of Study	Methodology/Data Analyzed with FEM	Values, Location of Load
Jakupović S. et al. 2014 [24]	Analysis of the abfraction lesions formation mechanism by the FEM	The authors evaluated stress in the lower premolar under static solicitations.	The intact tooth was scanned with a µCT 1076 SkyScan scanner (Kontich, Belgium). The images were reconstructed with the Nrecon (Skyscan) program into trans-axial sections. The additional reduction of input data was implemented, and the reconstruction of the 3D model was realized. Analyses of µCT section data were performed with the CTAn program (Skyscan), by using a 3D CAD tooth model (Matlab, Creo Parametric 1.0) with the Ansys Workbench 14.0 program.	200 N Paraxial 40°
Soares P.V. et al. 2015 [25]	Loading and composite restoration assessment of different non-carious cervical lesions morphologies—3D FEA	The impacts of various occlusal loadings on premolars with NCCLs (unrestored or restored with composite resins) were studied with 3D FEA.	The intact tooth was set in a contact scanner (MDX-40, Roland Co., Osaka, Japan). The 3D models of FEA were manufactured by simulating an intact tooth model, models with NCCL morphologies, and restored models of all types of lesions. For creating the volume of external and internal shapes of hard and soft tissues of tooth, the STL files were exported to CAD (Computer Assisted Design; Rhino3D 4.0, Rhinoceros, USA) software. NCCL morphologies and their restorations were obtained through Boolean operations. The models were examined with the biomechanical analysis software ANSYS 12.0 (Ansys Workbench 12.0.1, PA, EUA), with STEP format software, for pre-processing, processing, and post-processing. After the conversion mesh test, the solid quadratic tetrahedral components with ten nodes were utilized.	100 N Vertical Paraxial 45° buccal and palatal loading
Zeola I.F. et al. 2015 [26]	Influence of non-carious cervical lesions depth, loading point application and restora-tion on stress distribution pattern in lower pre-molars: A 2D FEA	The authors investigated the biomechanical behavior of lower premolars (the NCCL depth, load type, and restoration status) using FEA.	The intact extracted lower premolar scan images were studied with two-dimensional FEM. The scan data were exported to computer-aided design software (Autodesk Mechanical Desktop 6; Autodesk Inc., San Rafael, CA, USA) to generate the external contours of tooth structure and the polyether/polystyrene cylinder. The polyline module of CAD software was used for defining the tooth shapes, points, and line associations. The generated 2D FEA models were the healthy tooth, the three sizes of NCCL (small, medium, and deep), and the restored lesions. The obtained data were exported to CAE (computer-aided engineering) software (ANSYS 12.0, ANSYS Inc., Houston, TX, USA), using the *.IGES format. In the pre-processing phase, eight-noded isopara-metric plane elements were utilized (PLANE 183). The quantitative examination of strain (MPa) was determined at the enamel surface (on its upper wall), dentin (at the bottom wall), and dentin (on the lower wall) of the NCCLs.	100 N Oblique loads on buccal cusp: on internal occlusal surface; on outer buccal cusp surface
Jakupović S. et al. 2016 [27]	Biomechanics of cervical tooth region and non-carious cervical lesions of different morphology three dimensional finite element analysis	The authors examined, through 3D FEA, the stress distribution and the biomechanical behavior of two types of occlusal charging of hard dental tissues of the lower first premolar with abfraction.	Three-dimensional models of the lower first premolar with wedge-shaped lesions and saucer-shaped lesions (U lesions) were realized. Microcomputed tomography (µCT) X-ray images were obtained. A scanner (type SkyScan 1076 Kontich, Belgium) was used. The images were rebuilt into transaxial sections by using Nrecon and CTAn program software (SkyScan). A total of 570 horizontal sections were selected for the making of the 3D models. MATLAB program packages (MathWorks, Inc., Natick, MA, USA) and Creo Parametric 1.0 CAD software were used for obtaining the volumetric 3D CAD tooth model of the lower first premolar with abfraction lesions. FEM analysis of strain under functional and nonfunctional occlusal charging was realized with CTAn program 1.10 and ANSYS Workbench version 14.0.	200 N 40°
Zeola L.F. et al 2016 [28]	Effects of non-carious cervical lesion size, occlusal loading and restoration on biomechani-cal behaviour of premolar teeth	Investigation of the influence of NCCL dimensions, of restorations, and of occlusal loading direction on biomechanical behavior, as well as load tests and fracture toughness tests in the mandibular premolars, by FEA.	The intact extracted lower premolar scan images were studied with 2D FEM. The scan data were exported to computer-aided design software (Autodesk Mechanical Desktop 6; Autodesk Inc., San Rafael, CA, USA) to generate the external contours of tooth structure and the polyether/polystyrene cylinder. The polyline module of CAD software was used for defining the tooth shapes, points, and line associations. The generated 2D FEA models were the healthy tooth, the three sizes of NCCL (small, medium, and deep), and the restored lesions. The obtained data were exported to CAE (computer-aided engineering) software (ANSYS 12.0, ANSYS Inc., Houston, USA), using the *.IGES format. In the pre-processing phase PLANE 183 program was utilized. The quantitative examination of strain (MPa) was determined at the enamel surface (on its upper wall), dentine (at the bottom wall), and dentine of the NCCLs (on the lower wall).	100 N Loads on outer and inner slopes of buccal cusp
Costa L.S.V. et al 2017 [29]	Influence of the occlusal contacts in formation of abfraction lesions in the upper premolar	The authors used FEA to observe the repartition of the stress over the upper premolar, applying centric contact, working contact, and balancing contact occlusal loading, to realize a biomechanical comparison of these contacts with increased potential to induce NCCLs.	In the 3D model, a first maxillary premolar was used, along with their specific oral tissues (enamel, dentin, a periodontal ligament), and the fixation cylinder. The file was acquired with CAD software in STL format (Rhinoceros 5.0 McNeel, North America, OH, USA). The anatomic contours were designed using the Biocad method. Analysis software ANSYS 15.0, ANSYS Inc., Houston, TX, USA, was used. The meshes were generated by applying the tetrahedral elements and the convergence test. The mechanical properties were taken from the specialty literature and then inserted into the software for the static structural analysis. Axial and paraxial loads (40°) were reproduced for central occlusion, laterality on the occlusal slope of the oral working cusp, and interference contact to balance movement on the occlusal slope of the facial cusp. The shapes of dental elements were examined through the colorimetric scale for the determination of qualitative similarity in the simulated occlusions. The stress distribution over the course of the loadings was displayed by colorimetric graphs.	Axial loads Paraxial 40° loads on oral and buccal cusp
Machado, A.C. et al. 2017 [30]	Stress-strain analysis of premolars with non-carious cervical lesions: influence of restorative material, loading direction and mechanical fatigue.	The purpose of the study was to evaluate the stress and strain dispersion in upper pre-molars affected by NCCLs in accordance with factors such as restorative technique, direction of occlusal loads, and mechanical fatigue.	A 3D finite element analysis linear elastic investigation was conducted using the anatomical shape of the hard periodontal tissues of the tooth (pulp, dentin, enamel, periodontal ligament, and cortical and medullar bones). The 14 models were generated by Rhinoceros 3D software (Rhinoceros, Miami, FL, USA) and simulated the sound tooth, unrestored buccal saucer-shaped NCCLs, and restored NCCLs with different types of dental materials (resin-modified glass ionomer, flowable composite resin, conventional nanofilled composite resin, lithium disilicate glass ceramic, and conventional nanofilled composite resin core associated with a 0.5-mm lithium disilicate glass ceramic laminate). With the processing analysis software (ANSYS 12.0, Ansys Workbench 12.0.1, Canonsburg, PA, USA) and the Standard for the Exchange of Product Data (STEP) format, the preprocessing, processing, and postprocessing analyses were performed. A strain gauge was connected to the buccal surface of the analyzed tooth for the registration of tooth strains before and after cyclic loading (200,000 cycles, 50 N).	150 N Axial loads on both cusps Paraxial loads at a 45° angle on palatine cusp
Stănuşi, A. et al. 2019 [31]	Effects of occlusal loads in the genesis of non-carious cervical lesions—a finite element study	The size and repartition of stress in a maxillary first premolar of a 14-year-old patient suspected of having normal and heavy occlusal loads was studied.	A 3D model of the upper first premolar was realized through the CT images. Eight scenarios for the vertical loads and eight scenarios for the horizontal loads were used. To obtain the 3D model, the scanned images were converted by the MIMICS program and then processed in Abaqus/CAE so that they could be studied with FEA. The 3D virtual model contained the upper right first premolar tooth (enamel, dentine, and pulp) and their alveolar bone. The tooth was not presented rigidly in the bone, and between them existed the periodontal ligament with a 0.069 GPa elastic modulus. The FEA model presented tetrahedral elements (47,548 elements and 68,504 nodes). MIMICS Program and Abaqus/CAE were used. The 3D virtual model represented by the maxillary right first premolar and their alveoli were considered a parallelepiped.	180 N and 532 N Horizontal loads Vertical loads
Vuković, A.A. et al. 2019 [32]	Occlusal Stress Distribution on the Mandibular First Premolar—FEM Analysis.	The purpose of this paper was to realize an FEM investigation of the dispensation of stress and of the deformation in a lower first premolar by applying two types of loads.	Microcomputed tomography (µCT) and a scanner (SkyScan 1076 Kontich, Belgium) were used on an intact premolar tooth under two types of loading. So a volumetric 3D CAD tooth model was obtained by the utilization of MATLAB (MathWorks, Inc., Natick, MA, USA) program packages and Creo Parametric 1.0 CAD software. On the tooth model, a wedge-shaped abfraction lesion was formed, and then the models were transferred to the FEA software ANSYS Workbench (14.0). A FE-mesh of models was implemented. The properties of the tooth tissues were utilized. The model was fixed to simulate the periodontal ligament movement under 300µm loads and the contact points with neighboring teeth.	200 N Axial load Paraxial load
Stănuşi, A. et al. 2020 [33]	Analysis of stress generated in the enamel of an upper first premolar: a finite element study.	The aim of this article was to present the distribution and dimension of stress that appeared in the enamel of a maxillary first bicuspid tooth after exerting normal and excessive occlusal loadings.	A virtual model of the maxillary first premolar without any lesion was utilized to realize five models with distinct degrees of dental occlusal abrasion. The CT images of the maxillary premolar were transformed into 3D data by the MIMICS program (Materialise NV, Leuven, Belgium, 1992), and FEA was applied to study the resulting stresses. The scenarios obtained for occlusal loading of the 3D virtual model (14 in number) were investigated using the Abaqus/CAE program (ABAQUS Software, S.A.R.L., Versailles, France, 1994). The loads were exerted in the vertical and horizontal directions. The 3D models were tetrahedral elements with an average size of around 0.5 mm per surface.	180 N and 532 N Horizontal load Vertical load
Peres, T.S. et al. 2022 [34]	Influence of non-carious cervical lesions, bone attachment level, and occlusal load on the stress distribution pattern in maxillary premolars: finite element analysis.	The purpose of the study was to analyze the stress distribution design by using 3D FEA in upper premolars with and without NCCL prior to and after rehabilitation with composite resin. The impact of different occlusal loads on various bone attachment levels and tooth structure was also evaluated.	Nine CAD models were obtained after the intact models, through Rhinoceros 3D software (Rhinoceros, Miami, FL, USA). The design of the cervical area varied (intact tooth, wedge-shaped NCCL, restored NCCL with composite resin, but also normal level of bone, or with vertical and horizontal bone loss). For each model, vertical, buccal, and palatal loads were simulated. The analysis software ANSYS 12.0 (Ansys Workbench 12.0.1, Canonsburg, PA, USA). The Standard for the Exchange of Product Data (STEP) format was used, with preprocessing, processing/data calculation, and post-processing/analysis of stress dispersation. Stress values were collected after processing the models (maximum principal stress and minimum principal stress).	100 N Vertical load Buccal load Palatal load

**Table 7:** Description of included articles in the study (results and conclusion) [24,25,26,27,28,29,30,31,32,33,34].

No.	Author, Reference No., Year of Publishing	Main Results and Conclusions
1	Jakupović S. et al. [24] 2014	The action of occlusal loads (especially paraxial one) induces considerable stress in the cervical sub-superficial stratum of enamel. The stress values of the cervical area due to the paraxial loads are about five times higher in comparison with the superficial enamel layer.
2	Soares P.V. et al. [25] 2015	The morphology of NCCLs presented reduced consequences for the stress distribution design. The loading type and existence of composite resin restorations affected the biomechanical comportment of the maxillary premolars.
3	Zeola I.F. et al. [26] 2015	The depth of NCCLs is related to the intensification of the magnitude and the expansion of strain concentration. The application of loads on the external slope of the buccal cusps in mandibular premolars created a higher possibility of fracture in comparison with the application on the internal slope. Resin composite restoration of lesions augments the structural integrity and the biomechanics of roundabout NCCLs close to the healthy teeth.
4	Jakupović S. et al. [27] 2016	The exposure to occlusal loadings induces the progression of abfraction lesions. The type of action of these loadings impacts the stress intensity in the cervical area of teeth. The shape of abfraction lesions represents a major factor in the dispersal of internal stress. Underneath occlusal loads, V-shaped abfraction lesions presented notably higher stress accumulation in comparison with U-shaped abfraction lesions.
5	Zeola L.F. et al [28] 2016	The exposure to occlusal loadings induces the progression of abfraction lesions. The type of action of these loadings determines the stress intensity in the cervical area of teeth. The eccentric occlusal loads induced excessive calculated stress values in all hard tissues of the tested tooth models. The shape of the abfraction lesions also impacts the partition of internal stress in the hard tissues of the tooth.
6	Costa L.S.V. et al [29] 2017	For the quantitative comparison, the results appear in a table. The maximum values, which represent the concentrations of higher stress, are located in the enamel rather than the dentine. Eccentric contacts also present a higher possibility of progressing the abfraction lesions in the cervical area of the teeth and thus developing the size of tensile and shear stresses.
7	Machado, A.C. et al. [30] 2017	The presence of NCCLs associated with paraxial loads determined a higher stress concentration in the cervical area of the tooth. The paraxial load represented the major factor that impacted the biomechanical behavior of teeth affected by NCCLs. The restored NCCLs with composite resin alone or with ceramic laminates appears to be the optimum manner of direct rehabilitation since their biomechanical behaviors were almost identical with those of the sound teeth.
8	Stănuşi, A. et al. [31] 2019	The highest values of stress were situated in the oral cervical area, but may appear in other tooth regions too, where NCCLs are not commonly found. This fact implies that the genesis of cervical noncarious lesions is multifactorial.
9	Vuković, A.A. et al [32] 2019	Under the action of occlusal paraxial loads, the strain values were greater and more notable in the cervical area. In an abfraction lesion, under a paraxial load, the measured stress values were extremely high. Exposure to the stress of V abfraction lesions will determine their deepening. The deformation of the tooth below the paraxial load was approximately 10 times greater than below the axial load.
10	Stănuşi, A. et al. [33] 2020	Compressive stress predominated in the simulations of premolar models with horizontal occlusal abrasion. Both compressive and tensile stresses appeared in the simulations of the affected tooth models with oblique occlusal abrasions. The exceeding vertical and horizontal loads were prejudicial, regardless of their magnitude.
11	Peres, T.S. et al. [34] 2022	The presence of NCCL intensified the tensile stress concentration in the cervical third of the buccal surface. The tensile stress concentrations were the same in healthy and restored models. The stress level at the periodontal bone level was not affected by the presence or absence of NCCL, but it was associated with the occlusal load. The existence of NCCL contributed to the apparition of an excessive stress concentration in the tooth model cervical area, particularly when the oblique occlusal loads acted.

## Data Availability

Data supporting the reported results are contained within the article.
